# A natural polymorphism in Zika virus NS2A protein responsible of virulence in mice

**DOI:** 10.1038/s41598-019-56291-4

**Published:** 2019-12-27

**Authors:** Gines Ávila-Pérez, Aitor Nogales, Jun-Gyu Park, Silvia Márquez-Jurado, Francisco J. Iborra, Fernando Almazan, Luis Martínez-Sobrido

**Affiliations:** 1 Department of Microbiology and Immunology, University of Rochester Medical Center, 601 Elmwood Avenue, Rochester, New York 14642 USA; 2 Center for Animal Health Research, INIA-CISA, 28130 Valdeolmos Madrid, Spain; 3 Department of Molecular and Cell Biology, Centro Nacional de Biotecnología (CNB-CSIC), Universidad Autonóma de Madrid, 3 Darwin Street, 28049 Madrid, Spain

**Keywords:** Virology, Viral infection

## Abstract

Zika virus (ZIKV) infection is currently one of the major concerns in human public health due to its association with neurological disorders. Intensive effort has been implemented for the treatment of ZIKV, however there are not currently approved vaccines or antivirals available to combat ZIKV infection. In this sense, the identification of virulence factors associated with changes in ZIKV virulence could help to develop safe and effective countermeasures to treat ZIKV or to prevent future outbreaks. Here, we have compared the virulence of two related ZIKV strains from the recent outbreak in Brazil (2015), Rio Grande do Norte Natal (RGN) and Paraiba. In spite of both viruses being identified in the same period of time and region, significant differences in virulence and replication were observed using a validated mouse model of ZIKV infection. While ZIKV-RGN has a 50% mouse lethal dose (MLD_50_) of ~10^5^ focus forming units (FFUs), ZIKV-Paraiba infection resulted in 100% of lethality with less than 10 FFUs. Combining deep-sequencing analysis and our previously described infectious ZIKV-RGN cDNA clone, we identified a natural polymorphism in the non-structural protein 2 A (NS2A) that increase the virulence of ZIKV. Moreover, results demonstrate that the single amino acid alanine to valine substitution at position 117 (A117V) in the NS2A was sufficient to convert the attenuated rZIKV-RGN in a virulent Paraiba-like virus (MLD_50_ < 10 FFU). The mechanism of action was also evaluated and data indicate that substitution A117V in ZIKV NS2A protein reduces host innate immune responses and viral-induced apoptosis *in vitro*. Therefore, amino acid substitution A117V in ZIKV NS2A could be used as a genetic risk-assessment marker for future ZIKV outbreaks.

## Introduction

Zika virus (ZIKV) is an emerging mosquito-borne flavivirus, which became a global public concern due to its association with an increase in congenital microcephaly cases in Brazil in 2015^1^. ZIKV was first isolated in 1947 from a sentinel rhesus monkey in the Zika forest of Uganda^2^. ZIKV infection in humans was first described during a jaundice epidemic in Nigeria in 1954^3^ and only sporadic cases of ZIKV infection in humans have been reported in Africa and Asia over the last century^4–8^. It was not until 2007, when ZIKV caused the first outbreak on Yap Island in the Federated States of Micronesia with almost 75% of the population showing signs of infection^9^.

ZIKV was historically associated with a mild febrile illness, which is similar to that caused by other mosquito-borne diseases of public relevance such as Dengue virus (DENV), Yellow fever virus (YFV) or Chikungunya virus (CHIKV)^10^. The similarity of the clinical signs of ZIKV disease with those of DENV or CHIKV has interfered with ZIKV diagnosis and most probably underestimated the number of cases of ZIKV infections^11^. However, ZIKV has become a major human health concern due to its association with severe neurological complications, including an increased risk of Guillain-Barré syndrome in adults during the large outbreak in French Polynesia in 2013–2014^12,13^ and a dramatic increase in severe congenital malformations, including fetal microcephaly in neonates during the massive epidemic that emerged in Brazil in 2015^14–19^. Since then, millions of infected individuals have been reported in South and Central America, the Caribbean and the South of United States^20–22^. ZIKV is primarily transmitted through the bite of an infected *Aedes* spp. mosquito (*Aedes aegypti* and *Aedes albopictus*)^6,23–25^. However, non-vector ZIKV transmission has been reported to occur by vertical transmission from mother to child^14,26,27^ and by sexual transmission^25,28–30^. In addition, ZIKV RNA has been detected in body fluids including blood, urine, semen, saliva and breast milk, increasing the risk of a human to human transmission via direct contact with body fluids from infected people^31,32^.

ZIKV is a positive single-stranded RNA virus with a genome of about 10.8 kb that contains a cap structure at the 5′-end and a single open reading frame (ORF) flanked by two 5′ and 3′ untranslated regions (UTRs)^33,34^. The ORF encodes a single polyprotein of approximately 3,424 amino acids that is co- and post-translationally processed by viral and cellular proteases to produce three structural proteins, capsid (C), pre-membrane (prM) and envelope (E), and seven non-structural (NS) proteins (NS1, NS2A, NS2B, NS3, NS4A, NS4B and NS5)^33,34^. Phylogenetic analyses of ZIKV genomes have identified two major genetic lineages, African and Asian^35^. ZIKV strains from the recent epidemic in the Americas are phylogenetically related to the Asian lineage^36,37^. The molecular determinants of ZIKV evolution, spread, virulence, and disease have not been totally established. It has been postulated that ZIKV genome changes may have evolved to modify tissue tropism, becoming more neurotrophic^8,38–41^, and/or more efficiently transmitted to humans^8,32,42–44^, but the virulence factors associated with ZIKV pathogenesis remain unknown.

During the last years, extensive efforts have been made to develop *in vitro* and *in vivo* approaches to study ZIKV infection and pathogenesis, including reverse genetic systems^34^ and suitable animal models of infection^45^. In this sense, the emergence of ZIKV has promoted the rapid development of numerous reverse genetic approaches^34^, which constitute an essential tool for research to generate recombinant viruses containing specific substitutions to evaluate their contribution in viral replication or transcription, pathogenicity, virus-host interaction, viral tropism and transmissibility^46–48^. Recently, we have developed a reverse genetic approach based on the use of a bacterial artificial chromosome (BAC) to assemble the full-length cDNA of the viral genome of ZIKV Rio Grande do Norte Natal (RGN) strain (Brazil, 2015)^49^. On the other hand, animal models are essential to understand the biology and pathogenesis of ZIKV. It is known that infection of immunocompetent mice results in little to no virus production, and infected mice do not develop disease^50^. However, mice with deficiencies in the interferon (IFN) signaling pathway display enhanced susceptibility to infection by ZIKV and other flaviviruses, and they recapitulate many of the symptoms associated with infections in humans^45,51–54^. Therefore, these animal models are currently used to study ZIKV infection *in vivo*, including the development of new therapeutic approaches to combat and/or prevent ZIKV infections^24,45,50,52,55^.

In the present study, we have analyzed the virulence of ZIKV-RGN and Paraiba, two viral strains that have been identified from the recent outbreak in Brazil in 2015. Using the validated type-I IFN receptor deficient (IFNAR−/−) A129 mice model of ZIKV infection, we have observed that ZIKV-Paraiba is more virulent and replicates to higher levels compared to rZIKV-RGN. Combining deep-sequencing analysis and reverse genetic systems, we have identified a single amino acid polymorphism at position 117 in the viral protein NS2A responsible for the differences in virulence between both ZIKV strains. This polymorphism (A117V) in ZIKV NS2A affects host innate immune responses and viral-induced apoptosis, and, therefore, represents an important viral genetic marker for risk-assessment to prevent future ZIKV outbreaks.

## Results

### *In vitro* and *in vivo* properties of ZIKV-Paraiba and rZIKV-RGN

**Figure 1:**
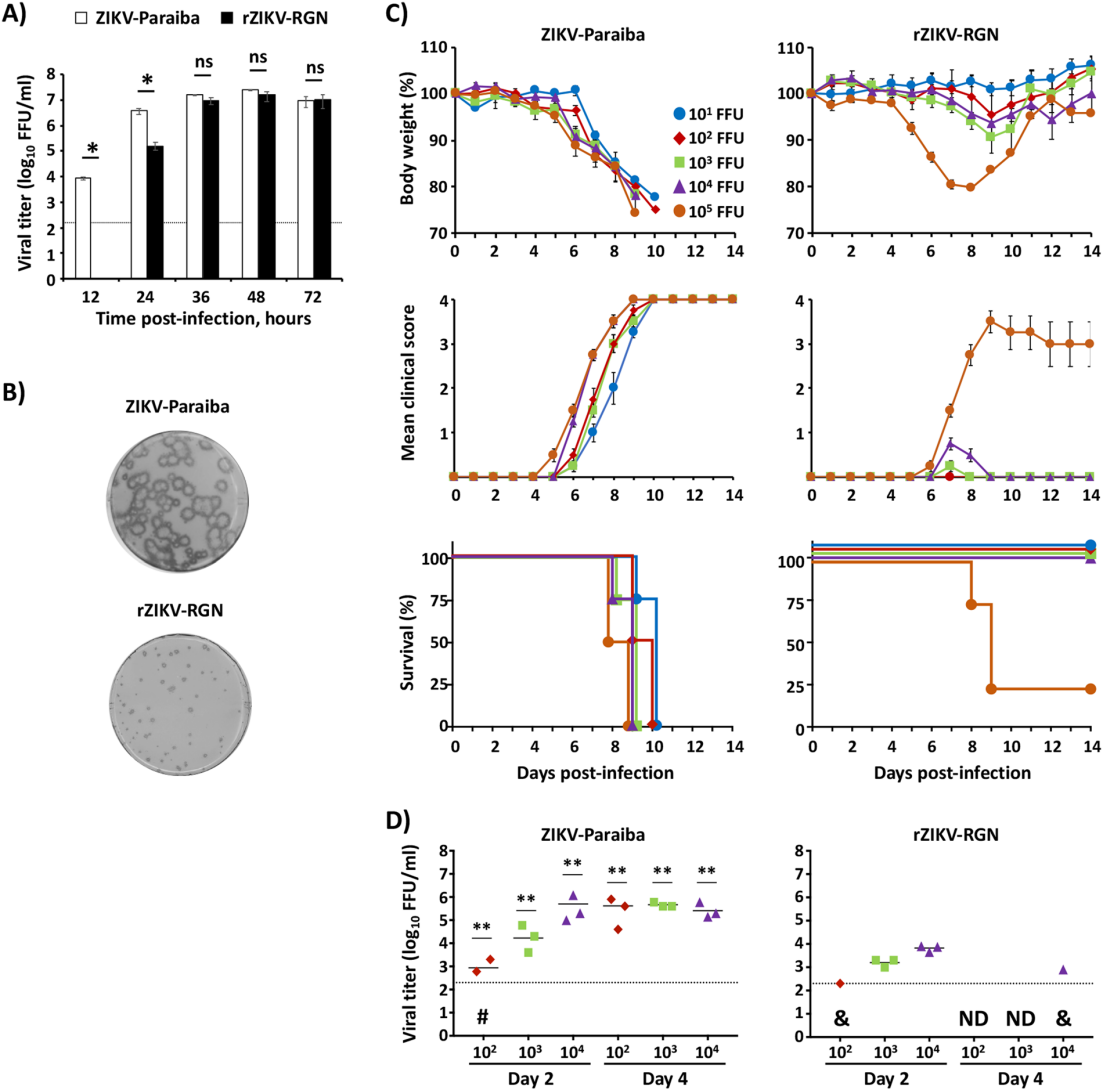
*In vitro* and *in vivo* characterization of ZIKV-Paraiba and rZIKV-RGN. (**A**) Viral growth kinetics: Vero cells (12-well plates, 0.5 × 10^6^ cells/well, triplicates) were infected with the ZIKV-Paraiba natural isolate (white columns) or with rZIKV-RGN (black columns) at MOI of 0.1 FFU/cell and viral titers in tissue cultured supernatants were determined at the indicated hpi by immunofocus assay (FFU/ml) using the E protein mAb 4G2. The black dotted line indicates the limit of detection (200 FFU/ml). Data represent the means +/− SD of the results determined in triplicate wells. *P < 0.05 using a Student’s t test. Ns, not significant (P > 0.05). (**B**) Plaque phenotype: Vero cells (6-well plates, 1 × 10^6^ cells/well) were infected with ~25–50 FFU of ZIKV-Paraiba (upper) or rZIKV-RGN (bottom) and overlaid with media containing agar. At 3 days post-infection, viral plaques were immunostained using the E protein mAb 4G2. **(C)** Morbidity and mortality: Six-to-seven-week-old female IFNAR−/− A129 mice (n = 4) were infected s.c. in the footpad with the indicated FFU of ZIKV-Paraiba (left panels) or rZIKV-RGN (right panels). Body weight (upper panels), clinical score (middle panels) and survival rates (lowers panels) were evaluated daily for 14 days. Error bars represent SD of the mean for each group of mice. Clinical signs were scored as described in the material and methods. (**D)** Viral titers: Six-to-seven-week-old female IFNAR−/− A129 mice (n = 6) were infected with the indicated FFU of ZIKV-Paraiba (left panel) or rZIKV-RGN (right panel) as described above. Mice were sacrificed at days 2 and 4 after infection (n = 3/time point) and viral titers in sera were determined by immunofocus assay (FFU/ml). Symbols represent data from individual mice and bars the geometric means of viral titers. ^#^Virus not detected in one mouse; ^&^virus not detected in two mice; ND, virus not detected. Dotted black lines indicate the limit of detection (200 FFU/ml). Differences in titers between ZIKV-Paraiba (left panel) and rZIKV-RGN (right panel) were analyzed by Student’s t test. **P < 0.01.

Recently, we have generated an infectious cDNA clone of ZIKV-RGN (pBAC-ZIKV-RGN) using a BAC approach^49^, from which infectious recombinant (r)ZIKV-RGN was successfully recovered after transfection of Vero cells^49^. The genome sequence of ZIKV-RGN was obtained from an infected fetus with microcephaly during the recent outbreak in Brazil in 2015^14^. Interestingly, when we compared the rZIKV-RGN with the contemporary ZIKV-Paraiba strain, isolated from a febrile female in the state of Paraiba (Brazil) in 2015, we observed significant differences *in vitro* and *in vivo* (Fig. 1) between both strains. Analysis of the growth kinetics reveled that both viruses replicated efficiently in Vero cells (Fig. 1A) reaching titers of approximately 10^7^ focus-forming units per ml (FFU/ml) at 48 hours post-infection (hpi). However, at early times post-infection (12 and 24 hpi) rZIKV-RGN had a significant delay in the replication kinetics compared to ZIKV-Paraiba. In addition, analysis of the plaque phenotype by immunostaining showed that at 3 days after infection, rZIKV-RGN produced smaller plaques than those of ZIKV-Paraiba (Fig. 1B).

To assess the pathogenicity *in vivo* of rZIKV-RGN and ZIKV-Paraiba, groups of six-to-seven-week-old female IFNAR−/− A129 mice (n = 4) were inoculated subcutaneously (s.c.) in the footpad with different doses (10^1^–10^5^ FFU/mouse) of ZIKV-Paraiba or rZIKV-RGN and monitored daily for signs of morbidity (body weight loss and clinical disease signs) and survival for 14 days (Fig. 1C). Notably, all mice infected with ZIKV-Paraiba suffered a rapid dose-dependent weight loss between days 5 to 10 (Fig. 1C, upper panel), showing clear neurological disease signs, including tremors, disorientation, hind limb weakness and severe paralysis (Fig. 1C, middle panel), and animals died or were euthanized between days 7–10 post-infection (Fig. 1C, lower panel). Surprisingly, only 10 FFU of ZIKV-Paraiba was sufficient to result in 100% of mortality (mouse lethal dose 50, MLD_50_ < 10 FFU). In contrast, and as we previously described^49^, only 75% of the mice infected with a high dose of 10^5^ FFU of rZIKV-RGN lost weight and succumbed to viral infection, resulting in a calculated MLD_50_ of ~7.5 × 10^4^ FFU (Fig. 1C, right panels). Remarkably, mice that succumbed to rZIKV-RGN infection presented similar disease symptoms than those observed with ZIKV-Paraiba. In the case of mice infected with 10^4^ FFU of rZIKV-RGN, some animals presented signs of illness (hunched posture and reduced movement) and lost some weight between days 7 and 9, but all of them recovered the initial body weight and survived viral infection (Fig. 1C, right panels). Mice infected with 10^1^–10^3^ FFU of rZIKV-RGN did not present clinical symptoms and all of them survived viral infection. In agreement with these observations, mice infected with ZIKV-Paraiba had significant higher viremia than mice infected with rZIKV-RGN on days 2 and 4 after infection (Fig. 1D), correlating the differences observed in morbidity and mortality (Fig. 1C) with the ability of these viruses to replicate *in vivo* (Fig. 1D).

### Genome sequence differences between ZIKV-Paraiba and rZIKV-RGN

To identify the virulence factors responsible for these dramatic differences in virulence between rZIKV-RGN and ZIKV-Paraiba, we first sequenced our laboratory stock of ZIKV-Paraiba (University of Rochester, UR) by deep-sequencing (Table S1). Although, the complete sequence of ZIKV-Paraiba was previously deposited in GenBank (KX280026), we did not know the passage history of our ZIKV-Paraiba. Several nucleotides differences between our laboratory and the reference ZIKV-Paraiba sequence were identified (Table S1). Interestingly, some of these differences corresponded with genetic variants or quasispecies, suggesting that ZIKV-Paraiba is a population of different genomes as previously described in the literature^56^. Based on these sequencing results, a consensus sequence for our ZIKV-Paraiba was determined, considering only the most frequent nucleotide variants (>50% of frequency) (Table S1, grey). Based on the consensus sequence, six non-synonymous nucleotides substitutions between our ZIKV-Paraiba and the reference KX280026 sequence were identified, resulting in 3 amino acid changes in the prM protein (E21G, T74A and S109P), one in the NS2A protein (A117V) and two in the NS3 protein (M334T and K587R) (Table S1, grey).

The genetic identity of our rZIKV-RGN was previously determined, showing 100% identity with the published ZIKV-RGN sequence (GenBank accession number KU527068)^49^. Comparative sequence analysis between rZIKV-RGN and our ZIKV-Paraiba (UR) showed 35 nucleotides differences between both viruses, resulting in 9 amino acid substitutions localized in the prM (E21G, T74A and S109P), NS1 (E146K and A233T), NS2A (A117V), NS3 (M334T and K587R) and NS4B (I240T) proteins (Table 1). One nucleotide substitution was present in the 3′ UTR (T10561C). Based on these data, we hypothesized that the 9 amino acid differences identified in the viral polyprotein or the nucleotide change in the 3′ UTR, or a combination of them, could be responsible of the differences in virulence and viral replication observed *in vivo* between RGN and Paraiba strains.

**Table 1:** Sequence differences between rZIKV-RGN and ZIKV-Paraiba University of Rochester (UR).

Nucleotide sequence			Amino acid sequence				Protein
Nucleotide position	rZIKV RGN	ZIKV Paraiba	Polyprotein position	Protein position^a^	rZIKV RGN	ZIKV Paraiba	
254	A	G	—	—	—	—	C
5*35*	*A*	*G*^*b*^	*143*	*21*	*E*	*G*	*prM*
6*93*	*A*	*G*	*196*	*74*	*T*	*A*	*prM*
7*98*	*T*	*C*	*231*	*109*	*S*	*P*	*prM*
8*36*	C	T	—	—	—	—	prM
923	G	A	—	—	—	—	prM
1,616	C	T	—	—	—	—	E
2,375	C	T	—	—	—	—	E
2,681	G	A	—	—	—	—	NS1
2,724	T	C	—	—	—	—	NS1
*2,925*	*G*	*A*	*940*	*146*	*E*	*K*	*NS1*
3,059	G	A	—	—	—	—	NS1
3,137	T	C	—	—	—	—	NS1
3,140	A	G	—	—	—	—	NS1
*3,186*	*G*	*A*	*1,027*	233	*A*	*T*	*NS1*
3,218	T	A	—	—	—	—	NS1
3,332	G	A	—	—	—	—	NS1
3,731	C	T	—	—	—	—	NS2A
*3,895*	*C*	*T*^*b*^	*1,263*	117	*A*	*V*	*NS2A*
5,438	A	G	—	—	—	—	NS3
*5,614*	*T*	*C*^*b*^	*1,836*	334	*M*	*T*	*NS3*
5,937	T	C	—	—	—	—	NS3
6,320	C	T	—	—	—	—	NS3
*6,373*	*A*	*G*	*2,089*	587	*K*	*R*	*NS3*
6,959	T	C	—	—	—	—	NS4B
7,451	A	C	—	—	—	—	NS4B
7,493	G	A	—	—	—	—	NS4B
*7,633*	*T*	*C*	*2,509*	240	*I*	*T*	*NS4B*
7,805	T	C	—	—	—	—	NS5
8,408	T	A	—	—	—	—	NS5
8,867	C	T	—	—	—	—	NS5
9,305	C	T	—	—	—	—	NS5
9,353	T	C	—	—	—	—	NS5
9,560	A	G	—	—	—	—	NS5
*10,561*^*c*^	*T*	*C*					3′*UTR*

Nucleotides differences that result in amino acid substitutions or changes localized in the 3′ untranslated region (UTR) are shown in italic. ^a^Amino acid changes in ZIKV proteins. ^b^Consensus sequence of ZIKV-Paraiba UR was determined considering only the more frequent (>50% of frequency) nucleotide variants shown in Table S1. ^c^Change in the 3′ UTR.

### Development and characterization of an infectious ZIKV-Paraiba cDNA clone

**Figure 2:**
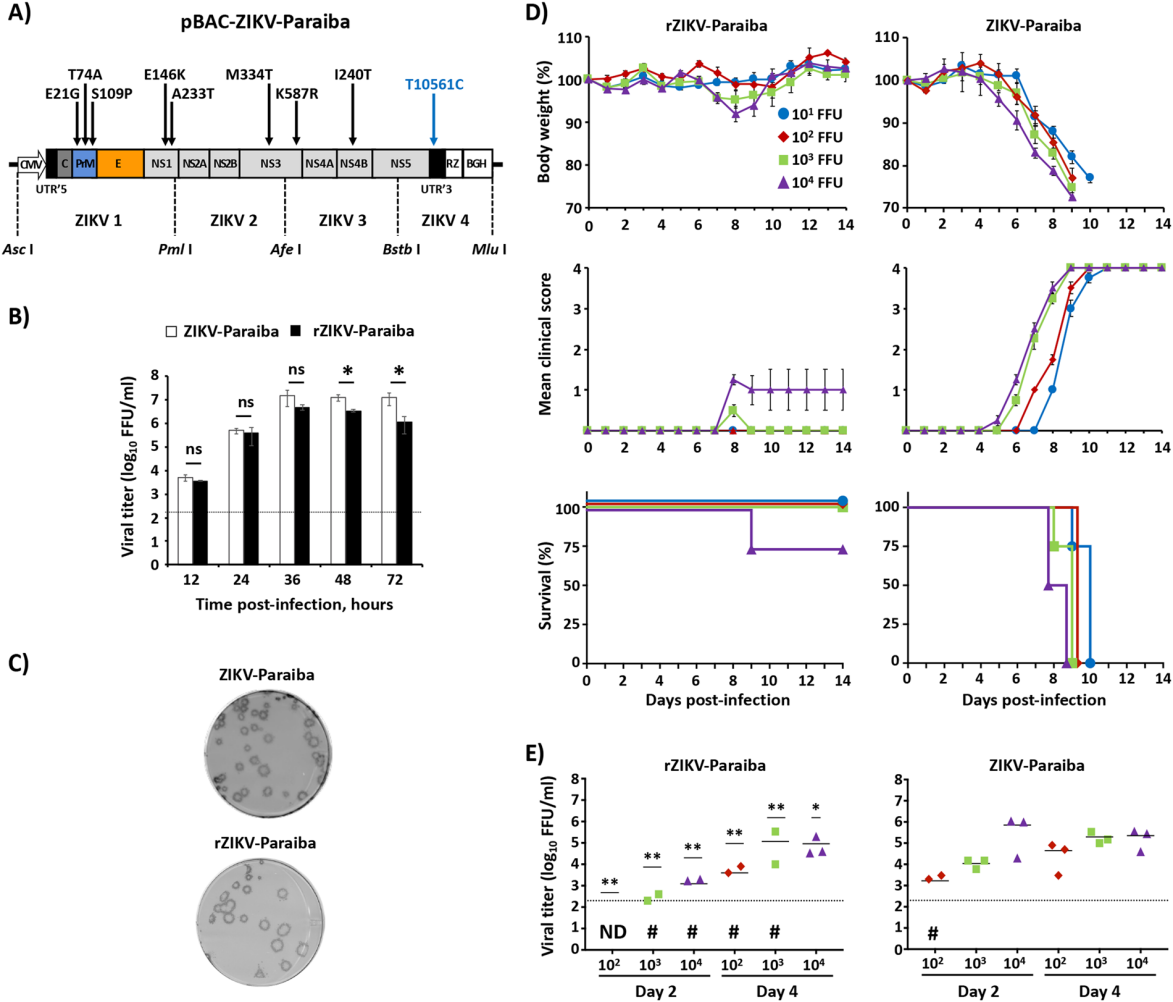
Generation and characterization of rZIKV-Paraiba. (**A**) Schematic representation of Paraiba infectious cDNA clone (pBAC-ZIKV-Paraiba): The diagram shows changes introduced in the rZIKV-RGN genome to generate the rZIKV-Paraiba cDNA clone, including eight non-silent mutations in the coding region (black arrows) and a single mutation in the 3′ UTR (blue arrow) (for more information see Table 1). Site directed mutagenesis was performed in the intermediate plasmids containing the ZIKV 1 to ZIKV 4 segments and assembled in the pBAC-ZIKV-RGN vector by replacing the corresponding fragment using the indicated restriction sites (*Asc* I, *Pml* I, *Afe* I, *BstB* I and *Mlu* I). The full-length cDNA is flanked at the 5′-end by the cytomegalovirus (CMV) promoter and at the 3′-end by the hepatitis delta virus (HDV) ribozyme (Rz) followed by the bovine growth hormone termination and polyadenylation sequences (BGH). The coding region from the structural (C, prM and E) and non-structural (NS1, NS2A, NS2B, NS3, NS4A, NS4B and NS5) proteins, as well as the 5′ and 3′ UTRs are illustrated. (**B)** Viral growth kinetics: Vero cells (12-well plates, 0.5 × 10^6^ cells/well, triplicates) were infected (MOI 0.1) with the natural isolate ZIKV-Paraiba (white columns) or with rZIKV-Paraiba (black columns) and the viral titers in tissue cultured supernatants were determined at the indicated times post-infection by immunofocus assay (FFU/ml). The black dotted line indicates the limit of detection (200 FFU/ml). Data represent the means +/− SD of the results determined in triplicate wells. *P < 0.05 using a Student’s t test. Ns, not significant (P > 0.05). (**C**) Plaque phenotype: Vero cells (6-well plate format, 1 × 10^6^ cells/well) were infected with ~25–50 FFU of ZIKV-Paraiba (upper) or rZIKV-Paraiba (bottom) and overlaid with media containing agar. At 3 days post-infection, viral plaques were immunostained using the E protein mAb 4G2. (**D**) Morbidity and mortality: Six-to-seven-week-old female IFNAR−/− A129 mice (n = 4) were infected s.c. in the footpad with the indicated FFU of rZIKV-Paraiba (left panels) or ZIKV-Paraiba (right panels). The body weight (upper panels), clinical score (middle panels) and survival rate (lower panels) were evaluated daily for 14 days. Error bars represent SD of the mean for each group of mice. Clinical signs were scored as indicated in material and methods. (**E**) Viral titers: Six-to-seven-week-old female IFNAR−/− A129 mice (n = 6) were infected with the indicated FFU of rZIKV-Paraiba (left panel) or ZIKV-Paraiba (right panel) as described above. Mice were sacrificed 2 or 4 days post-infection (n = 3/time point) and viral titers in sera were determined by immunofocus assay (FFU/ml). Symbols represent data from individual mice and bars the geometric means of viral titers. ^#^Virus not detected in one mouse; ND, virus not detected. Dotted black lines indicate the limit of detection (200 FFU/ml). Differences in titers between rZIKV-Paraiba (left panel) and ZIKV-Paraiba (right panel) were analyzed by Student’s t test. **P < 0.01. *P < 0.05.

To identify the virulence factors associated with ZIKV-Paraiba, we first generated an infectious cDNA clone of ZIKV-Paraiba (pBAC-ZIKV-Paraiba) using the reverse genetic approach previously described to generate rZIKV-RGN^49^ (Fig. 2). To that end, we used site directed mutagenesis to introduce the non-synonymous substitutions (Fig. 2A, black arrows), with the exception of A117V in the NS2A protein that is present in ZIKV-Paraiba, into our previously described pBAC-ZIKV-RGN^49^. Likewise, the T10561C change in the 3′ UTR was also introduced (Fig. 2A, blue arrow). We did not introduce the amino acid substitution A117V in the pBAC-ZIKV-Paraiba infectious clone because this substitution causes a conservative amino acid change (A to V) in approximately 50% of ZIKV-Paraiba (Table S1). In addition, analysis of 684 publicly available ZIKV sequences showed that 98.1% of ZIKV strains contain an alanine amino acid residue at position 117 in the NS2A protein (https://www.viprbrc.org/brc/home.spg?decorator = flavi_zika). A rZIKV-Paraiba was successfully recovered after transfection of the pBAC-ZIKV-Paraiba in Vero cells. When growth kinetics on Vero cells of the natural ZIKV-Paraiba isolate and the rZIKV-Paraiba were compared, we found that both viruses replicated efficiently (Fig. 2B) and have similar plaque phenotype (Fig. 2C). Nonetheless, as seen with other rZIKVs^56,57^, viral titers were slightly reduced as compared to the natural ZIKV isolate at later time points (Fig. 2B), which can be due to the loss of viral quasispecies diversity as it has been widely discussed^58,59^. Importantly, the plaque size of rZIKV-Paraiba was similar to that of the natural ZIKV-Paraiba (Fig. 2C), suggesting that the nine amino acid substitutions and the change in the 3′ UTR introduced in the infectious rZIKV-RGN clone to generate rZIKV-Paraiba resulted in increased viral replication and plaque phenotype in culture cells. Taken together, these results demonstrate that we were able to rescue a rZIKV-Paraiba with similar *in vitro* characteristics than the natural viral isolate.

To determine whether the rescued rZIKV-Paraiba was pathogenic *in vivo*, groups of six-to-seven-week-old female IFNAR−/− A129 mice (n = 4) were inoculated s.c. in the footpad with different doses (10^1^–10^4^ FFU/mouse) of rZIKV-Paraiba or the natural ZIKV-Paraiba isolate and monitored daily for morbidity (body weight loss and clinical disease signs) and mortality for 14 days (Fig. 2D). As previously showed, mice infected with only 10 FFU of natural ZIKV-Paraiba began to lose weight at days 5 or 6 post-infection and all of them succumbed to viral infection between days 8–10 post-infection (Fig. 2D, right panels). In contrast, rZIKV-Paraiba was highly attenuated as compared to the natural ZIKV-Paraiba isolate (Fig. 2D, left panels). Mice infected with 10^1^–10^2^ FFU of rZIKV-Paraiba did not show any signs of disease. In the case of animals infected with 10^3^ FFU of rZIKV-Paraiba, only one mouse presented slightly weight loss and survived viral infection. Mice infected with 10^4^ FFU of rZIKV-Paraiba presented evident signs of infection, and 3 of 4 mice showed weight loss between days 6 and 8 post-infection, but only one of them succumbed to viral infection at day 9 post-infection (MLD_50_ > 1 × 10^4^ FFU) (Fig. 2D, left panels). To analyze rZIKV-Paraiba viremia, groups of six-to-seven-week-old female IFNAR−/− A129 mice (n = 6) were infected with different doses (10^2^–10^4^ FFU/mouse) of rZIKV-Paraiba or the natural ZIKV-Paraiba isolate, and viral titers in mouse sera were analyzed at days 2 and 4 post-infection (Fig. 2E). Viral titers of ZIKV-Paraiba in mice sera were significantly higher than those of rZIKV-Paraiba at all the doses and times analyzed (Fig. 2E). Altogether, these data indicated that although the 9 amino acid substitutions and the single nucleotide T10561C change in the 3′ UTR introduced in the rZIKV-RGN infectious clone to generate rZIKV-Paraiba increased viral replication *in vitro* (Figs. 1A and 2B) and *in vivo* (Figs. 1D and 2E) compared to rZIKV-RGN, rZIKV-Paraiba was attenuated *in vivo*, showing comparable virulence than rZIKV-RGN, demonstrating that these mutations are not responsible for the phenotypic differences between the natural isolate ZIKV-Paraiba and rZIKV-RGN.

### rZIKV-Paraiba containing the amino acid substitution A117V in the NS2A protein is highly virulent *in vivo*

**Figure 3:**
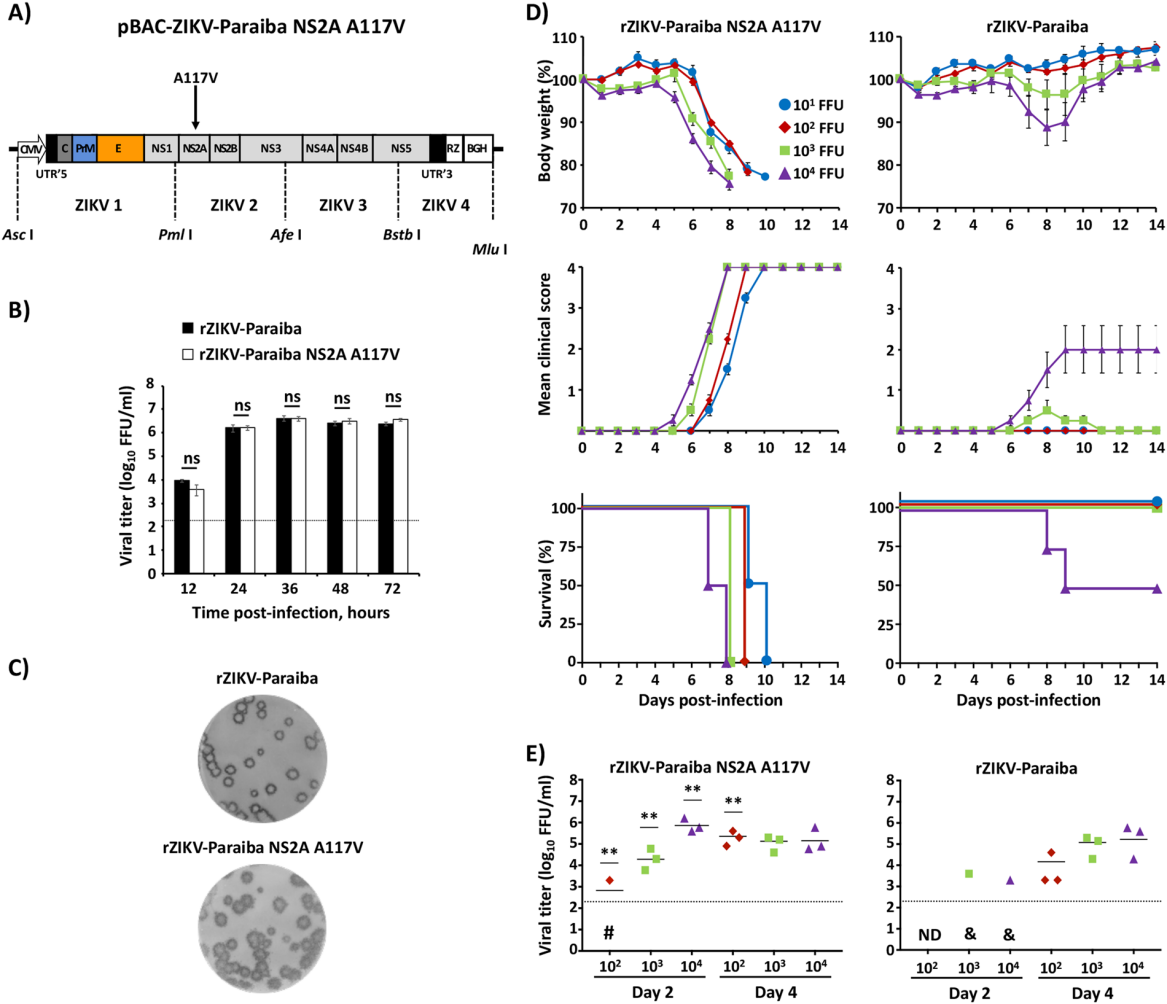
Characterization of rZIKV-Paraiba containing a valine at position 117 in NS2A (rZIKV-Paraiba NS2A A117V). (**A**) Schematic representation of Paraiba NS2A A117V infectious cDNA clone (pBAC-ZIKV-Paraiba NS2A A117V): The diagram shows the alanine (**A**) to valine (V) substitution in the NS2A protein (black arrow) introduced in the pBAC-ZIKV-Paraiba (Fig. 2A) to generate the pBAC-ZIKV-Paraiba NS2A A117V. Site directed mutagenesis was performed in the intermediate plasmids containing the ZIKV 2 segment of Paraiba and assembled in the pBAC-ZIKV-Paraiba using the *Pml* I and *Afe* I restriction sites. Symbols and abbreviations are the same than those described in Fig. 2A. (**B**) Viral growth kinetics: Vero cells (12-well plates, 0.5 × 10^6^ cells/well, triplicates) were infected (MOI 0.1) with rZIKV-Paraiba (black columns) or rZIKV-Paraiba NS2A A117V (white columns) and viral titers in tissue culture supernatants were determined at the indicated times post-infection by immunofocus assay (FFU/ml) using the E protein mAb 4G2. The black dotted line indicates the limit of detection (200 FFU/ml). Data represent the means +/− SD of the results determined in triplicate wells. Ns, not significant (P > 0.05). (**C**) Plaque phenotype: Vero cells (6-well plate format, 1 × 10^6^ cells/well) were infected with ~25–50 FFU of rZIKV-Paraiba (upper) or rZIKV-Paraiba NS2A A117V (bottom) and overlaid with media containing agar. At 3 days post-infection, viral plaques were immunostained using the E protein mAb 4G2. (**D**) Morbidity and mortality: Six-to-seven-week-old female IFNAR−/− A129 mice (n = 4) were infected s.c. in the footpad with the indicated FFU of rZIKV-Paraiba NS2A A117V (left panel) or rZIKV-Paraiba (right panels). The body weight (upper panels), clinical score (middle panels) and survival rate (lower panels) were evaluated daily for 14 days. Error bars represent SD of the mean for each group of mice. Clinical signs were scored as described in material and methods. (**E**) Viral titers: Six-to-seven-week-old female IFNAR−/− A129 mice (n = 6) were infected with the indicated FFU of rZIKV-Paraiba NS2A A117V (left panel) or rZIKV-Paraiba (right panel) as described above. Mice were sacrificed at days 2 or 4 post-infection (n = 3/time point) and viral titers in sera were determined by immunofocus assay (FFU/ml). Symbols represent data from individual mice and bars the geometric means of viral titers. ^#^Virus not detected in one mouse; ^&^virus not detected in two mice; ND, virus not detected. Dotted black lines indicate the limit of detection (200 FFU/ml). Differences in titers between rZIKV-Paraiba NS2A A117V (left panel) and rZIKV-Paraiba (right panel) were analyzed by Student’s t test. **P < 0.01.

During the deep-sequencing analysis of ZIKV-Paraiba, we identified several genetic variants in our laboratory ZIKV-Paraiba stock (Table S1), which could be responsible of the differences observed in virulence between ZIKV-Paraiba and rZIKV-RGN (Fig. 1C). Importantly, Miner *et al*. described an increase in the frequency of a C to T substitution at position 3,895 in eye-, spleen-, and brain- derived viruses from ZIKV-Paraiba infected mice^60^. We noted that this single nucleotide substitution (C to T) results in the conservative amino acid substitution (A117V) in ZIKV NS2A protein, the same amino acid change identified in our initial deep sequencing analysis (Table S1) that was not included in the construction of the infectious rZIKV-Paraiba clone (Fig. 2). To explore the possibility that this NS2A A117V substitution was responsible for the differences in virulence between the natural and recombinant ZIKV-Paraiba, an infectious clone of rZIKV-Paraiba containing the additional NS2A A117V change (pBAC-ZIKV-Paraiba NS2A A117V) was generated by site-directed mutagenesis (Fig. 3A). The rZIKV-Paraiba NS2A A117V was successfully recovery from Vero cells, and its replication *in vitro* was compared to that of rZIKV-Paraiba (Fig. 3B,C). Importantly, rZIKV-Paraiba NS2A A117V grew similarly to rZIKV-Paraiba in Vero cells, with no statistical differences in the viral growth kinetics at any of the time points analyzed (Fig. 3B). Moreover, rZIKV-Paraiba NS2A A117V and rZIKV-Paraiba produced plaques with similar size after 3 days of infection (Fig. 3C).

To investigate whether rZIKV-Paraiba NS2A A117V was virulent *in vivo*, groups of six-to-seven-week-old female IFNAR−/− A129 mice (n = 4) were inoculated s.c. (footpad) with different doses (10^1^–10^4^ FFU/mouse) of rZIKV-Paraiba NS2A A117V or rZIKV-Paraiba (Fig. 3D). Mice were monitored daily for 14 days for signs of morbidity (body weight loss and clinical disease signs) and mortality (Fig. 3D). Interestingly, and similar to mice infected with ZIKV-Paraiba (Figs. 1C and 2D), all mice infected with rZIKV-Paraiba NS2A A117V rapidly lost weight in a dose-dependence manner, showing strong signs of infection, necessitating euthanasia between days 7–10 post-infection, with a calculated MLD_50_ < 10 FFU (Fig. 3D, left panels). In contrast, and as previously shown in Fig. 2D, rZIKV-Paraiba was highly attenuated, with 100% of mice surviving the 10^1^ to 10^3^ FFU infectious doses and 50% of them succumbing to viral infection with 10^4^ FFU (MLD_50_ 10^4^ FFU) (Fig. 3D, right panels). To demonstrate that virulence correlates with viral replication, six-to-seven-week-old female mice were infected with 10^2^–10^4^ FFU of rZIKV-Paraiba NS2A A117V or rZIKV-Paraiba (Fig. 3E). In agreement with the morbidity and mortality results, mice infected with rZIKV-Paraiba NS2A A117V (Fig. 3E, left panel) presented statistically higher viremia than mice infected with rZIKV-Paraiba at day 2 post-infection (Fig. 3E, right panel), which was comparable to replication of the natural ZIKV-Paraiba isolate (Fig. 1D, left panel and Fig. 2E, right panel). However, mice infected with 10^3^ and 10^4^ FFU of rZIKV-Paraiba (Fig. 3E, right panel) produced high viremia at day 4 post-infection, reaching titers of approximately 10^5^ FFU/ml, which were similar to those obtained for the rZIKV-Paraiba NS2A A117V (Fig. 3D, left panel) or ZIKV-Paraiba infections (Figs. 1D and 2E). Altogether, these data support that the single amino acid substitution A117V in ZIKV NS2A protein is responsible for the differences in virulence between ZIKV-Paraiba natural isolate and rZIKV-RGN (Fig. 1).

### Analysis of clones of ZIKV-Paraiba containing NS2A A117 or V117

**Figure 4:**
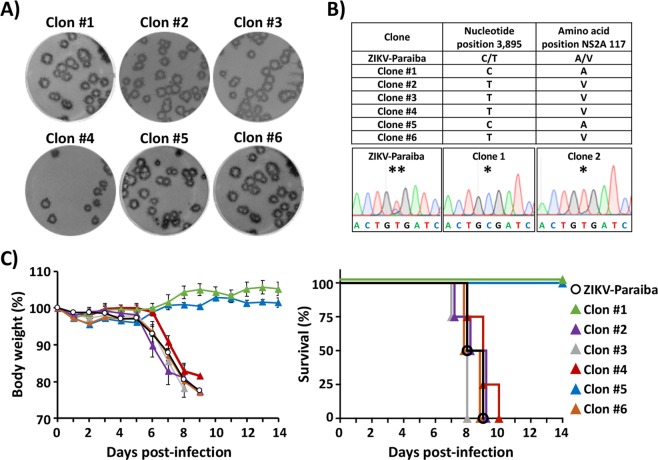
Characterization of plaque-purified ZIKV-Paraiba clones: Six representative clones (#1 to #6) of ZIKV-Paraiba were isolated by plaque purification on Vero cells and further characterized. (**A**) Plaque phenotype: Vero cells (6-well plate format, 1 × 10^6^ cells/well) were infected with ~25–50 FFU of each individual ZIKV-Paraiba clone and overlaid with media containing agar. At 3 days post-infection, viral plaques were immunostained using E protein mAb 4G2. (**B**) Sequence of ZIKV-Paraiba individual clones: RNA from Vero cells infected (MOI 0.1) with the ZIKV-Paraiba plaque-purified clones #1 to #6 or the parental ZIKV-Paraiba natural isolate were used to amplify the region 2,667–4,110, covering NS2A amino acid 117, by RT-PCR. PCR products were gel purified and sequenced. Results indicate the nucleotide found at position 3,895 together with the corresponding amino acid at position 117 in NS2A. Representative chromatograms of ZIKV-Paraiba natural isolate, clone#1 and clone #2 are shown. **Indicates the presence of two peaks at nucleotide 3,895 for the ZIKV-Paraiba natural isolate. *Indicates a single nucleotide at position 3,895 for ZIKV-Paraiba clones 1 (C) or 2 (T). (**C**) Morbidity and mortality: Six-to-seven-week-old female IFNAR−/− A129 mice (n = 4) were infected s.c., in the footpad with 10^3^ FFU of the indicated individual ZIKV-Paraiba clones, and body weight (left panel) and survival (right panel) were evaluated daily for 14 days. Error bars represent SD of the mean for each group of mice.

Our deep-sequencing analysis results indicate that ZIKV-Paraiba exist as a population of different genomes or quasispecies (Table S1). To further determine whether the presence of V117 in ZIKV NS2A was responsible of virulence, we isolated 6 individual clones (#1 to #6) of ZIKV-Paraiba by plaque purification on Vero cells (Fig. 4). Our plaque assay results showed that all the individual isolated ZIKV-Paraiba clones have similar plaque phenotype in Vero cells (Fig. 4A). However, sequencing analysis determined that clones #1 and #5 have a C (NS2A A117) while clones #2, #3, #4 and #6 have a T (NS2A V117) at position 3,895 (Fig. 4B), correlating with the proportion previously determined by deep-sequencing of the natural isolate ZIKV-Paraiba (40% C and 60% T; Table S1). To determine the virulence of these plaque-purified ZIKV-Paraiba clones *in vivo*, groups of six-to-seven-week-old female IFNAR−/− A129 mice (n = 4) were infected s.c. in the footpad with 10^3^ FFU/mice of the individual isolated clones (#1 to #6) and monitored for body weight loss and survival (Fig. 4C) for 14 days. As internal control for these studies, mice were infected with 10^3^ FFU of ZIKV-Paraiba. Interestingly, only ZIKV-Paraiba plaque-purified clones containing NS2A V117 (clones #2, #3, #4 and #6) were pathogenic in mice, showing strong signs of neurological disease with hind limb paralysis (data not shown) and animals died or were euthanized between days 7–10 post-infection (Fig. 4C). In contrast, mice inoculated with ZIKV-Paraiba plaque-purified clones #1 and #5 containing NS2A A117 survived viral infection, without disease signs (data not shown) or body weight lost (Fig. 4C), demonstrating that ZIKV NS2A V117 is responsible, at least in part, for the *in vivo* pathogenicity of the natural ZIKV-Paraiba.

### Virulence of a rZIKV-RGN NS2A A117V

**Figure 5:**
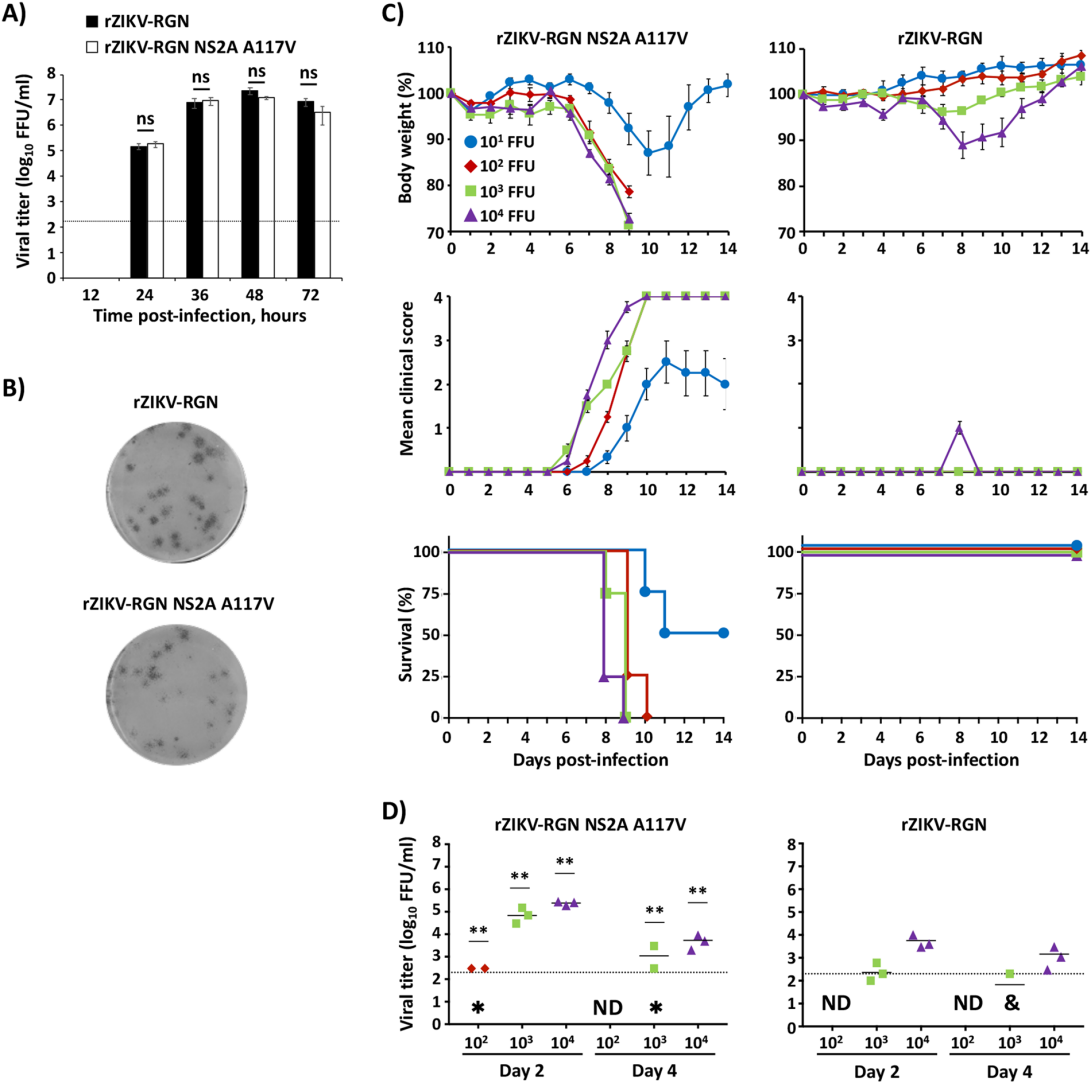
Characterization of rZIKV-RGN containing a valine (V) at position 117 in NS2A (rZIKV-RGN NS2A A117V). (**A**) Viral growth kinetics: Vero cells (12-well plates, 0.5 × 10^6^ cells/well, triplicates) were infected (MOI 0.1) with the rZIKV-RGN (black columns) or rZIKV-RGN NS2A A117V (white columns) and viral titers in tissue culture supernatants were determined at the indicated times post-infection by immunofocus assay (FFU/ml) using the E protein mAb 4G2. The black dotted line indicates the limit of detection (200 FFU/ml). Data represent the means +/− SD of the results determined in triplicate wells. Ns, not significant (P > 0.05). (**B**) Plaque phenotype: Vero cells (6-well plate format, 1 × 10^6^ cells/well) were infected with ~25–50 FFU of rZIKV-RGN (upper) or rZIKV-RGN NS2A A117V (bottom) and overlaid with media containing agar. At 3 days post-infection, viral plaques were immunostained using the E protein mAb 4G2. (**C**) Morbidity and mortality: Six-to-seven-week-old female IFNAR−/− A129 mice (n = 4) were infected s.c. in the footpad with the indicated FFU of rZIKV-RGN NS2A A117V (left panels) or rZIKV-RGN (right panels). The body weight (upper panels), clinical score (middle panels) and survival rate (lower panels) were evaluated daily for 14 days. Error bars represent SD of the mean for each group of mice. Clinical signs were scored as described in material and methods. (**D**) Viral titers: Six-to-seven-week-old female IFNAR−/− A129 mice (n = 6) were infected with the indicated FFU of rZIKV-RGN NS2A A117V (left panel) or rZIKV-RGN (right panel) as described above. Mice were sacrificed at 2 or 4 days post-infection (n = 3/time point) and the viral titers in sera were determined by immunofocus assay (FFU/ml). Symbols represent data from individual mice and bars the geometric means of viral titers. ^#^Virus not detected in one mouse; ^&^virus not detected in two mice; ND, virus not detected. Dotted black lines indicate the limit of detection (200 FFU/ml). Differences in titers between rZIKV-RGN NS2A A117V (left panel) and rZIKV-RGN (righy panel) were analyzed by Student’s t test. **P < 0.01.

Next, we evaluated whether the single NS2A substitution A117V could be sufficient to increase the virulence of rZIKV-RGN. To that end, we generated an infectious clone containing the substitution C to T at position 3,895 (NS2A A117V) in the rZIKV-RGN genome (pBAC-ZIKV-RGN NS2A A117V) (Fig. 5). After rescue of the recombinant virus in Vero cells, we compared the *in vitro* phenotype of rZIKV-RGN NS2A A117V and rZIKV-RGN (Fig. 5A,B). The rZIKV-RGN NS2A A117V grew similarly to rZIKV-RGN, showing no statistical differences in the viral titers at any of the time points analyzed (Fig. 5A). Likewise, the plaque phenotype was similar for rZIKV-RGN A117V and rZIKV-RGN (Fig. 5B), indicating that the single amino acid substitution A117V in the NS2A protein does not have an effect in viral replication *in vitro*. Next, we assessed the contribution of NS2A A117V in virulence and replication *in vivo* (Fig. 5C,D). Groups of six-to-seven-week-old female IFNAR−/− A129 mice (n = 4) were inoculated s.c. (footpad) with different doses (10^1^–10^4^ FFU/mouse) of rZIKV-RGN NS2A A117V or rZIKV-RGN, and animals were monitored for body weight loss (Fig. 5C, upper panels), clinical signs (Fig. 5C, middle panels) and survival (Fig. 5C, lower panels) for 14 days. Mice infected with 10^2^ to 10^4^ of rZIKV-RGN NS2A A117V rapidly lost weight and all of them succumbed to viral infection by day 8–10 post-infection (Fig. 5C, left panels). Notably, 2 of 4 mice infected with 10 FFU of rZIKV-RGN NS2A A117V showed clear signs of infection, lost weight rapidly and died by day 10–11 post-infection (Fig. 5C, left panels). The remaining two mice displayed weight loss and lethargy, although both survived viral infection (Fig. 5C, left panels). Based in this data, the MLD_50_ determined for rZIKV-RGN NS2A A117V was ~10 FFU. As expected, mice infected with 10^4^ FFU/mouse of rZIKV-RGN had reduced morbidity sings and all of them recovered and survived viral infection (Fig. 5C, right panels). Remarkably, the single substitution A117V in ZIKV NS2A results in a marked increase virulence with 3 to 4 logs of difference in the MLD_50_ between rZIKV-RGN (MLD_50_ ~7.5 × 104 FFU, Figs. 1 and 5) and rZIKV-RGN NS2A A117V (~10 FFU, Fig. 5). Notably, the pathogenesis observed with rZIKV-RGN NS2A A117V resemble that observed with the natural ZIKV-Paraiba isolate (Fig. 1). In addition, to evaluate whether the virulence observed *in vivo* correlates with viral replication, groups of six-to-seven-week-old female IFNAR−/− A129 mice (n = 6) were inoculated with 10^2^ to 10^4^ FFU of rZIKV-RGN NS2A A117V or rZIKV-RGN and viremia was determined at 2 and 4 days post-infection (Fig. 5D). The rZIKV-RGN NS2A A117V replicated with higher titers than rZIKV-RGN, with differences of 2–3 or 1 log at days 2 and 4 post-infection, respectively. Altogether, these results demonstrate that the substitution A117V in ZIKV NS2A contributes significantly to viral pathogenesis and viremia, and represents a new and important ZIKV virulence factor.

### Substitution A117V in ZIKV NS2A protein reduces host innate immune responses and viral-induced apoptosis in tissue culture cells

**Figure 6:**
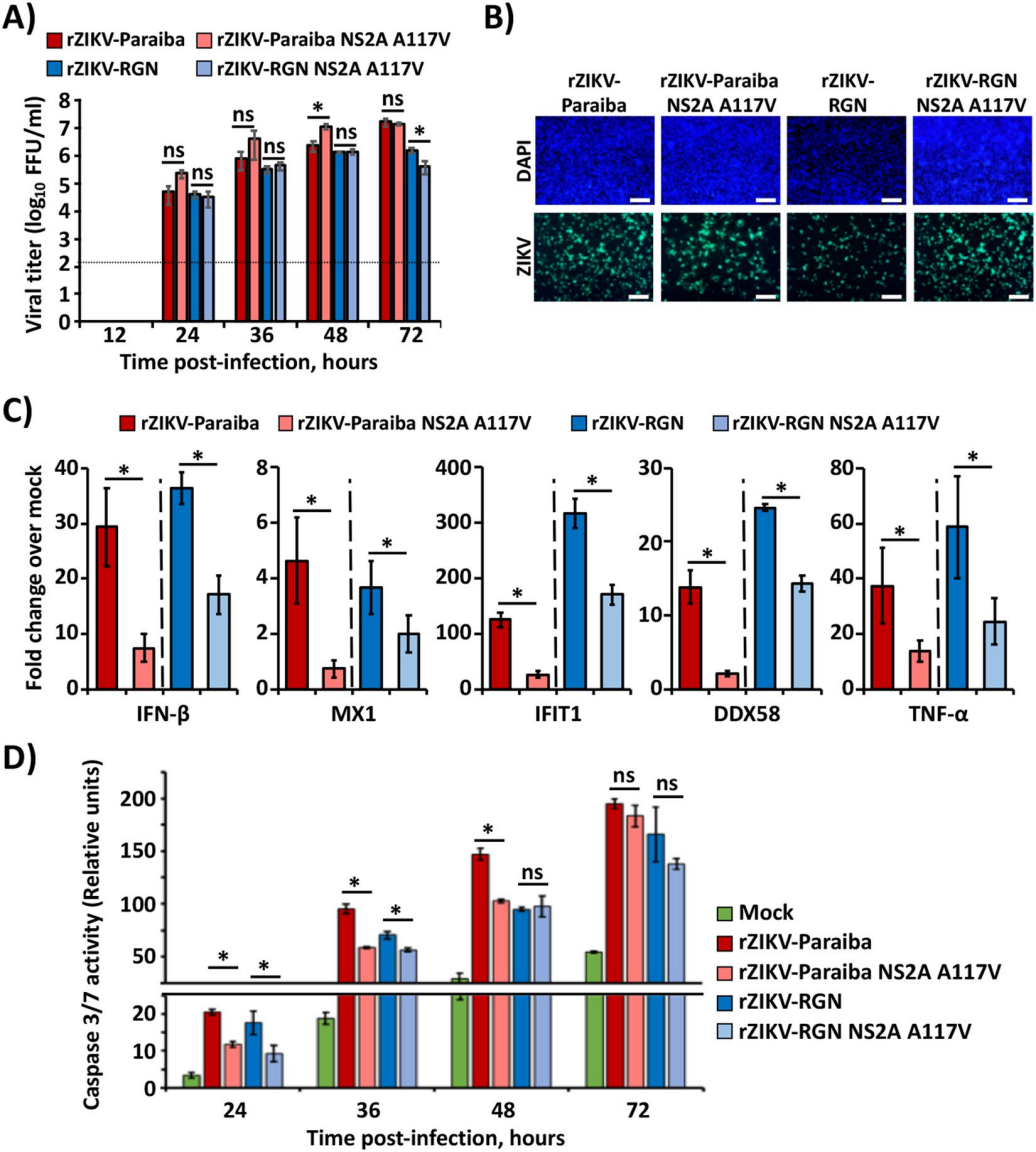
Effect of ZIKV NS2A A117V substitution in viral infection. (**A**) Viral growth kinetics: A549 cells (12-well plates, 5 × 10^5^ cells/well, triplicates) were infected (MOI 0.1) with the indicated viruses and viral titers in tissue cultured supernatants were determined at the indicated hpi by immunofocus assay (FFU/ml) using the E protein mAb 4G2. The black line indicates the limit of detection (200 FFU/ml). Data represent the means and SD of the results determined in triplicate wells. *P < 0.05 using a Student’s t test. ns, not significant (P > 0.05). (**B)** and (**C**) Effect of NS2A A117V substitution in induction of innate immune responses: A549 cells (12-well plates, 5 × 10^5^ cells/well, triplicates) were mock-infected or infected (MOI 3) with the indicated viruses. At 24 hpi viral infections were evaluated by immunofluorescence using the E protein mAb 4G2 (Scale bars = 2 mm) (**B**) and the RNA expression levels of IFN-β, MX1, IFIT1, DDX58 and TNF-α were quantified by RT-qPCR (**C**). Fold expression changes were calculated relative to mock-infected cells. Gene expression levels were normalized to the levels of the housekeeping gene H2B and to the ZIKV genome (WT *vs*. NS2A A117V for each viral strain). Data represent the means and SD values. Fold change analysis was done using the ΔΔCt method. *P < 0.05 using a Student’s t test. (**D)** Effect of NS2A A117V substitution in ZIKV-induced apoptosis: A549 cells (24-well plates, 2.5 × 10^5^ cells/well, triplicates) were mock-infected (green) or infected (MOI 0.1) with the indicated viruses and Caspase 3/7 activity was analyzed using the Caspase Glo 3/7 kit at the indicated hpi. Caspase activity values were relativized to mock-infected cells at 24 hpi. Data represent the means and SD values of the results determined in triplicates. *P < 0.05 using a Student’s t test. ns, not significant (P > 0.05).

Flavivirus NS2A protein is a membrane-associated protein that has been involved in the modulation of host innate immune responses^61–64^. To determine whether substitution A117V in ZIKV NS2A protein has an immunomodulatory role, the innate immune responses were analyzed in cells infected with rZIKV Paraiba or RGN carrying V117 or A117 in the NS2A protein (Fig. 6). Vero cells used to analyzed the growth kinetics of these rZIKVs (Figs. 1A, 2B, 3B and 5A) are known to be deficient in IFN production^65^. For this reason, A549 cells, an innate immune competent cell line derived from human lung adenocarcinoma, were chosen to assess innate immune responses. Furthermore, A549 cells have been previously reported to support efficient replication of ZIKV, leading to the production of IFN, IFN stimulated genes (ISGs) and pro-inflammatory cytokines^43^. Firstly, we evaluated whether rZIKV-Paraiba, rZIKV-Paraiba NS2A A117V, rZIKV-RGN or rZIKV-RGN NS2A A117V could infect and grow efficiently in A549 cells (Fig. 6A). Data indicate that all viruses were able to replicate in A549 cells and the polymorphism in NS2A did not significantly affect, at least at early times post-infection, the production of infectious virus. Then, A549 cells were infected (MOI 3) with same rZIKVs (Fig. 6B), and the expression levels of IFN-β, several ISGs, including myxovirus resistance protein 1 (MX1), IFN-induced protein with tetratricopeptide repeats 1 (IFIT1) and DExD/-box helicase 58 (DDX58), and human tumor necrosis factor alpha (TNF-α) were evaluated by quantitative RT-PCR (RT-qPCR) at 24 hpi (Fig. 6C). In agreement with previous data in the literature^43^, ZIKV infection resulted in the induction of IFN-β, ISGs (Mx1, IFIT1 and DDX58) and TNF-α (Fig. 6C). However, a significantly decreased in mRNA expression levels of these genes were observed in cells infected with rZIKV-Paraiba or rZIKV-RGN containing a V117 in NS2A (rZIKV-Paraiba NS2A A117V and rZIKV-RGN NS2A A117V) compared with the viruses containing A117 in NS2A (rZIKV-Paraiba and rZIKV-RGN) (Fig. 6C). These results suggest that amino acid substitution A117V in ZIKV NS2A can modulate antiviral responses by negatively regulating the expression of innate immune genes.

Systematically, we observed an increase in the induction of cytopathic effect (CPE) during infection with rZIKV-Paraiba or rZIKV-RGN as compared with rZIKV-Paraiba NS2A A117V or rZIKV-RGN NS2A A117V (data not shown). In addition, previous studies have shown that ZIKV infection can trigger apoptotic cell death in human neuronal progenitor^66^ or in epithelial A549^43^ cells. To confirm this observation, we evaluated whether the amino acid substitution A117V in ZIKV NS2A was involved in the regulation of apoptosis during viral infection by measuring caspase 3/7 activity (Fig. 6D**)**. To that end, A549 cells were mock infected or infected (MOI 0.1) with rZIKV-Paraiba, rZIKV-Paraiba NS2A A117V, rZIKV-RGN or rZIKV-RGN NS2A A117V, and caspase 3/7 activity was analyzed at 24, 36, 48 and 72 hpi. As expected, ZIKV infection resulted in activation of caspase 3/7 activity, starting at 24 hpi (Fig. 6D). Importantly, we observed a significant increase in caspase 3/7 activity during infection with rZIKV-Paraiba or rZIKV-RGN as compared with rZIKV-Paraiba NS2A A117V or rZIKV-RGN NS2A A117V, respectively, at early times post-infection (Fig. 6D). However, these differences were not significant at 72 hpi or even at 48 hpi for rZIVK-RGN (Fig. 6D). These results suggest that substitution A117V in ZIKV NS2A prevents or delays the induction of apoptosis, which correlate with the differences observed in the levels of TNF-α induction (Fig. 6C). Altogether, these data indicated that in tissue culture cells, ZIKV NS2A V117 modulates antivirals responses and delay the induction of apoptosis during viral infection.

## Discussion

Determining the viral factors associated with ZIKV disease is critical to understand and combat ZIKV infection, as well as to predict future potential viral outbreaks. In this study, we have compared the virulence of two closely related ZIKV strains, RGN and Paraiba, from the recent outbreak in Brazil in 2015. Despite of both viruses being identified at the same time period and region, significant differences in viral replication and virulence were observed *in vitro* and *in vivo* using a validated mouse model of ZIKV infection (Fig. 1). Mice infected with only 10 FFU of ZIKV-Paraiba showed clear disease signs, had a dramatic weight loss, and all of them succumbed to viral infection with a calculated MLD_50_ < 10 FFU. In contrast, only mice infected with 10^5^ FFU of rZIKV-RGN showed clear signs of infection, loss significant body weight and succumbed to viral infection, with a calculated MLD_50_ of ~7.5 × 10^4^ FFU (Fig. 1). Genome sequencing analysis of both viruses showed the presence of 9 amino acid changes in the coding region (prM: E21G, T74A and S109P; NS1: E146K and A233T; NS2A: A117V; NS3: M334T and K587R; NS4B: I240T) and one nucleotide substitution at the 3′ UTR (T10561C) (Table 1). In a first approach, we introduced all these substitutions, with the exception of NS2A A117V, in our previously described rZIKV-RGN infectious clone^49^ to generate a rZIKV-Paraiba (Fig. 2). Notably, although these changes improved viral replication *in vitro*, they did not have a significant effect in viral pathogenesis *in vivo* (Fig. 2). Surprisingly, we found that the single amino acid change A117V in NS2A was sufficient to recapitulate the *in vivo* pathogenic phenotype of ZIKV-Paraiba in the presence (Fig. 3) or in the absence (Fig. 5) of the remaining amino acids changes identified in our genome sequencing analysis.

Interestingly, the *in vitro* growth kinetic and plaque phenotype characteristics of rZIKV-Paraiba were, at least in part, attributed to the effect of 8 amino acid substitutions in the coding region (prM: E21G, T74A and S109P; NS1: E146K and A233T; NS3: M334T and K587R; NS4B: I240T) and/or the nucleotide substitution in the 3´UTR (Fig. 2). The individual contribution for each change, as well as other synonymous changes between ZIKV-Paraiba and rZIKV-RGN identified in our sequencing analysis (Table 1) were not directly addressed in this study and they could also play an important role in viral replication and/or virulence.

There is an urgent need for comparative analysis between ZIKV strains to identify virulence factors associated with viral pathogenesis. To date, a limited number of studies have been performed comparing disease caused by different ZIKV isolates, suggesting lineage-specific differences involved in viral pathogenesis^67–69^. Interestingly, Yuan *et al*. provided experimental evidences that a single amino acid substitution (S139N) in ZIKV prM acquired just prior to the outbreak in French Polynesia, contributes to the increased neurovirulence of contemporary ZIKV strains^41^. In addition, Liu *et al*. demonstrated that a single amino acid change (A188V) in ZIKV NS1 protein promotes the acquisition of ZIKV by *Aedes* mosquitoes from an infected host, and, therefore, the virus prevalence in mosquitoes^70^. Interestingly, these two amino acid changes (S139N in prM and A188V in NS1) are present in the viral strains used in our study, suggesting that in addition to these two evolutionary adaptations, other specific genome differences or polymorphisms that contribute to ZIKV pathogenesis could explain the recent outbreak of ZIKV.

The polymorphism NS2A V117 has been identified in 5 ZIKV sequences deposited in public databases. One of them was isolated from a patient with cutaneous rash, pain, and fever during the outbreak in Brazil in 2015 (HS/2015 Bahia_01, KX520666.1). The remaining 4 ZIKV were isolated from *Aedes aegypti* mosquitoes including a virus in Malaysia in 1966 (P6–740, KX377336.1) and 3 in Mexico (Mex_1_7/2016, KX446951.2; Mex_1_44/2016, KX856011; and 31 N/2018, MH900227.1). Nonetheless, as previously described in other ZIKV studies^27,56,71^, we observed a population of genetic variants or quasispecies in our ZIKV-Paraiba stock (Table 1). Substitution NS2A A117V was detected with a frequency of 53% in our ZIKV-Paraiba stock (Table S1). However, previous studies showed that this substitution was present with a frequency of 3%^56^ or 12%^60^ in other ZIKV-Paraiba stocks. Notably, it was detected with a frequency of 15.8% in H/PF/2013, a clinical strain isolated in French Polynesia in 2013^72^, suggesting that this mutation could be present in other ZIKV strains with a low frequency. Importantly, in our plaque purification studies (Fig. 4), we have been able to isolate individual ZIKV-Paraiba clones with NS2A A117 or V117 and demonstrate how NS2A V117 is responsible for virulence of ZIKV-Paraiba *in vivo* (Fig. 4). These studies were further supported when we generated the rZIKV-Paraiba (Figs. 2 and 3) or rZIKV-RGN containing NS2A V117 (Fig. 5). Surprisingly, rZIKV-RGN was isolated from an infected fetus with microcephaly in Brazil in 2015, but the full-length sequence identified an alanine at position 117 in the viral NS2A protein^14^. It is possible that, similar to our ZIKV-Paraiba, a low percentage of ZIKV-RGN NS2A V117 was present that would not allow the identification of this viral quasispecie during the sequencing process, in which only the majoritarian sequences are usually annotated. It is worth noting that ZIKV-RGN was not isolated from this microcephaly case^14^, making difficult to further evaluate this possibility in this and/or future studies.

Flavivirus NS2A protein is a multifunctional membrane-associated protein that has been involved in viral RNA synthesis^73,74^, virus assembly^75–77^, and immunomodulation of innate immune responses^61–64^ during flavivirus infection. To date, the role(s) of ZIKV NS2A in viral infection and pathogenesis are not well understood, although by homology with the rest of flaviviruses, ZIKV NS2A could have similar functions. Recently, ZIKV NS2A, but not DENV NS2A, has been involved in the disruption of mammalian cortical neurogenesis by degrading adherents junctions proteins^39^. Also, we previously identified an alanine-to-valine substitution at residue 175 (A175V) in ZIKV NS2A, which is important for viral RNA synthesis and pathogenesis *in vivo*^49^. In this study, we demonstrated that a single amino acid substitution (A117V) in ZIKV NS2A has a significant impact in viral pathogenesis and replication *in vivo*. Although we have analyzed the presence of ZIKV in the sera of three infected mice, future and more exhaustive studies will be required to determine if substitution A117V in NS2A affects viral replication in other organs and/or tissues where ZIKV has been shown to replicate, such as brain, placenta, and/or testis^14,24,27,78^. Likewise, a major effort to evaluate the role of ZIKV NS2A on viral replication and virulence is clearly needed.

IFN is responsible to induce a potent antiviral state by eliciting the upregulation of hundreds of ISGs conferring protection in both infected cells and neighboring non-infected cells. However, viruses have developed sophisticated strategies to inhibit or delay the IFN response and/or the antiviral activity of ISGs, including Flaviviruses^61–64^ and ZIKV^51^. Our results show that virulent rZIKVs carrying V117 in the NS2A protein induced significantly less IFN-β, ISGs (Mx1, IFIT1, DDX58) and TNF-α compared to the attenuated rZIKVs containing an A117. Also, a delay in the induction of CPE was observed in rZIKV-RGN and rZIKV-Paraiba containing the A117V change in NS2A, which correlates with a delay in the induction of apoptosis in infected cells (Fig. 6). Notably, and similar to our studies, early induction of apoptosis is generally associated with viral attenuation *in vivo*^79–81^, since apoptosis is a powerful antiviral host mechanism to limit viral replication, triggered by the IFN response^82^. This could represent, at least in part, the mechanism underlying the increased pathogenesis observed in the natural ZIKV-Paraiba or rZIKV (RGN and Paraiba) containing the A117V substitution in NS2A. It worth noting that although we have used type-I IFN (IFN-α/β) receptor deficient (IFNAR−/−) A129 mice in our studies, these mice retain intact type-II IFN (IFN-γ) responses^24^, which are involved in the induction of innate immune responses and apoptosis in multiples cells lines and may contribute to the control of viral replication and spread^82^. In fact, several studies using IFNAR−/− A129 mice have described the role of IFN-γ in viral infection^24,83^, reporting that IFN-γ contributes to the control of viral replication and spread.

In summary, we have identified a single amino acid change (A117V) in ZIKV NS2A that dramatically increases virulence *in vivo*. This, as well as other amino acid changes in ZIKV NS2A protein^49^, open the possibility of targeting ZIKV NS2A for the rational design of life attenuated vaccines for the prophylactic treatment of ZIKV infections. Likewise, these studies also open the feasibility of targeting ZIKV NS2A protein with small molecule compounds for the therapeutic treatment of ZIKV infection.

## Materials and Methods

### Cells and viruses

Vero African green monkey kidney epithelial (ATCC, CCL-81) and human A549 (ATCC, CCL-185) cells were maintained at 37 °C with 5% CO_2_ in Dulbecco’s modified Eagle’s medium (DMEM) supplemented with 5% fetal bovine serum (FBS), 2 mM L-glutamine, 100 units/ml penicillin and 100 μg/ml streptomycin (1x PSG).

ZIKV strain Paraiba (Brazil, 2015) was kindly provided by Dr. Stephen Dewhurst (Department of Microbiology and Immunology, University of Rochester Medical Center). The rZIKV-RGN was previously described^49^. Virus stocks were propagated in Vero cells and titrated by plaque assay as previously described^49^.

### Viral genome sequencing

Viral RNA was isolated from purified virions using a QIAamp viral RNA minikit (Quiagen). Deep-sequencing was conducted at the University of Rochester Genomics Research Center using Illumina MiSeq (Illumina, San Diego, CA, USA) as previously described^49^.

### Construction of ZIKV infectious cDNA clones

Infectious cDNA clones pBAC-ZIKV-Paraiba, pBAC-ZIKV-Paraiba NS2A A117V and pBAC-ZIKV-RGN NS2A A117V were generated using our previously described pBAC-ZIKV-RGN infectious cDNA clone^49^. To generate the pBAC-ZIKV-Paraiba, 8 non-silent mutations in the viral coding region (prM: E143G, T196A and S231P; NS1: E940K, and A1027T; NS3: M1826T, and K2089R; NS4B: I2509T) and a single nucleotide substitution (T10561C) in the 3′ UTR (Table 1) were introduced by site-direct mutagenesis in intermediate plasmids containing ZIKV fragments ZIKV1 to ZIKV4^49^. The modified cDNA fragments were replaced in the pBAC-ZIKV-RGN using selected restriction sites^49^. Additional silent nucleotide mutations were introduced in order to generate a *BsrB* I (position 2,921 A to G) and *Sap* I (position 3,167 A to G; 3,171 T to A; and 3,172 C to G) restriction sites as genetic markers. To generate pBAC-ZIKV-Paraiba NS2A A117V and pBAC-ZIKV-RGN NS2A A117V, a single amino acid change (A117V) at NS2A was introduce in the pBAC-ZIKV-Paraiba and pBAC-ZIKV-RGN, respectively. All plasmid constructs were confirmed by sequencing (ACGT, Inc.), propagated in DH10B *E. coli* (Gibco/BRL), and prepared using the Qiagen large-construct kit (Qiagen Inc.) following the manufacturer’s specifications. Primers sequences for the construction of the different plasmids are available under request.

### Plasmid transfection and virus recovery

Infectious viruses were recovered from the BAC cDNA clones as previously described^49^. Briefly, subconfluent monolayers of Vero cells (6-well plate format, 1 × 10^6^ cells/well) were transiently transfected with 4 μg of the BAC cDNA clones using 12 μl of Lipofectamine 2000 (Invitrogen). After 6 h, transfection media was removed, replaced with fresh viral growth medium (DMEM supplemented with 2% FBS and 1x PSG) and cells were incubated a 37 °C. When CPE was evident (60–80%), between 4 to 6 days, tissue culture supernatants were collected and stored at −80 °C. The identity of all the recover viruses was confirmed by sequencing (ACGT, Inc.) with specific primers. Briefly, viral RNA was purified using the RNeasy minikit (Qiagen Inc.) from infected Vero cells. Reverse transcription (RT) was performed using the High-capacity cDNA transcription kit (ThermoFisher Scientific) with 1ug of purified RNA and random hexamer primers. The cDNAs were amplified using the expand high fidelity PCR kit (Roche) and sequenced using specific primers, available under request.

### Virus titration

Viral titers were determined by immunofocus assay. Briefly, subconfluent monolayers of Vero cells (96-well plate format, 5 × 10^4^ cells/well, triplicates) were infected with 50 μl of 10-fold serial dilutions of virus-containing tissue culture supernatants samples for 90 min at 37 °C. After viral absorption, virus inoculum was removed, and the cell monolayers were overlaid with 100 μl of viral growth media containing 1% microcrystalline cellulose (Avicel, Sigma-Aldrich). After 36 hpi, cells were fixed with 4% formaldehyde for 1 h at room temperature and the overlays removed. Cells were then permeabilized with 0.5% Triton X-100 in PBS for 15 min at room temperature, blocked with 2.5% bovine serum albumin (BSA) in PBS, and used for immunofluorescence with the pan-flavivirus E protein monoclonal antibody (mAb) 4G2 (BEI resources; NR-50327) and a secondary AlexaFluor488-conjugated goat anti-mouse IgG (Invitrogen). Viral titers were expressed as FFU/ml, determined by the average number of E-positive foci at the highest dilutions in triplicates.

### Plaque assay and immunostaining

Subconfluent monolayers of Vero cells (6-well plate format, 1 × 10^6^ cells/well) were infected with 0.5 ml of viruses for 90 min at 37 °C. Then, 2 ml of viral growth media supplemented with 0.6% Agar Noble (Difco) and 1% DEAE-Dextran (Sigma-Aldrich) was added to each well. After 3 days of incubation at 37 °C, cells were fixed with 4% formaldehyde for 1 h and the overlays removed. Cells were then permeabilized with 0.5% Triton X-100 in PBS for 15 min at room temperature and prepared for immunostaining using the pan-flavivirus E protein mAb 4G2. Viral plaques were visualized using Vectastin ABC kit and DAB HRP substrate (Vector Laboratories Inc.), following the manufacturer’s instructions.

### Plaque purification of ZIKV-Paraiba clones

A plaque assay of ZIKV-Paraiba was performed as describe above. Six representative viral plaques were isolated and grown in Vero cells. When CPE was evident (60–80%), between 2 to 3 days post-infection, tissue culture supernatants were collected and stored at −80 °C. Viral titers were determined by immunofocus assay in Vero cells as described above. The presence of A117 or V117 in the NS2A protein was confirmed by sequencing (ACGT, Inc.).

### Virus grown kinetics

Subconfluent monolayers of Vero or A549 cells (12-well plate format, 0.5 × 10^6^ cells/well, triplicates) were infected (MOI 0.1) with the indicated viruses. After 90 min of virus absorption at 37 °C, the virus inoculum was removed and infected cells were overlaid with 2 ml of fresh viral growth media and incubated a 37 °C. At the indicated times post-infection, aliquots of tissue culture supernatants were collected and viral titers were determined by immunofocus assay in Vero cells as described above. The mean value and SD were calculated using Microsoft Excel software.

### Mice experiments

IFNAR−/− A129 mice (The Jackson Laboratory) were bred and maintained in the animal care facility at University of Rochester under specific pathogen-free conditions. All animal protocols were approved by the University of Rochester Committee of Animal Resources and complied with the recommendations in the Guide for the Care and Use of Laboratory Animals of the National Research Council^84^. Six-to-seven-week-old female IFNAR−/− A129 mice (n = 4) were anesthetized intraperitoneally with a mixture of ketamine (100 μg per gram of body weight) and xylazine (20 μg per gram of body weight) and then infected s.c. in the footpad with the indicated doses of ZIKV-Paraiba, rZIKV-Paraiba, rZIKV-Paraiba NS2A A117V, rZIKV-RGN or rZIKV-RGN NS2A A117V diluted in PBS in a final volume of 50 μl. Mice were monitored daily for clinical signs of ZIKV infection (lethargy, disorientation, hind limb weakness and severe paralysis), body weight loss and mortality for 14 days. Clinical signs were scored as follows: 0, healthy; 1, lightly sick (hunched back, lethargy and disorientation); 3, reduced mobility (hind limb weakness); and 4, severe paralysis and mortally. Mice showing more than 25% loss of their initial body weight or presenting severe hind limb paralysis were considered to have reached the experimental endpoint and were humanely euthanized. To correlate sings of disease with virus replication, six-to-seven-week-old female (n = 6) were infected as described above and bled at days 2 (n = 3) and 4 (n = 3) after viral infection. Blood was incubated a room temperature for 30 min, clarified by centrifugation at 1,500 × g for 20 min, and the supernatant was immediately stored at −80 °C. Viral titers in serum were determined by immunofocus assay as previously described. GraphPad Prism software was used to determine the geometric mean titers and the Reed and Muench method to determine the MLD_50_.

### Cellular gene expression and viral (v)RNA synthesis

Cellular gene expression and vRNA synthesis were evaluated by RT-qPCR. Total intracellular RNA from mock-infected or infected (MOI of 3) A549 cells with rZIKV-Paraiba, rZIKV-Paraiba NS2A A117V, rZIKV-RGN and rZIKV-RGN NS2A A117V was purified at 24 hpi using the RNeasy minikit (Qiagen Inc.), following manufacturer’s specifications. Total cDNA was synthesized from 250 ng of the purified RNA using random hexamer primers and the high-capacity cDNA Transcription kit (Applied Biosystems, ThermoFisher Scientific). Amplified cDNA was used to evaluate cellular gene expression using specific TaqMan assays (Applied Biosystems, ThermoFisher Scientific) for TNF-α (Hs00174128_m1), IFN-β (Hs01077958_s1), MX1 (Hs00895608-m1), IFIT1 (Hs03027069_s1), and DDX58 (Hs01061436_m1). To quantify the level of vRNA, a custom TaqMan assay specific for ZIKV RNA was used^49^. In all cases, histone H2B (TaqMan assay Hs00868438_s1, Applied Biosystems, ThermoFisher Scientific) was used as a reference housekeeping gene to normalize the differences in RNA sampling. Data were acquired with a 7500 qPCR system (Applied Biosystems, ThermoFisher Scientific) and further analyzed with ABI PRISM 7500 software v2.06. All quantifications were done using the cycle threshold (2^−ΔΔCT^) method^85^.

### Caspase 3/7 activity assay

Quantification of caspase 3/7 activity was performed using the Caspase Glo 3/7 assay kit (Promega) following the manufacturer’s specifications. Briefly, subconfluent monolayers of A549 cells (24-well plate format, 0.25 × 10^6^ cells/well, triplicates) were mock-infected or infected (MOI 0.1) with the indicated viruses. After 1 h of virus absorption, the viral inoculum was removed and 1 ml of fresh viral growth media was added. At the indicated times post-infection, cells were harvested in the tissue culture supernatants and frozen until their analysis. Cells lysates were incubated 1:1 with the Caspase-Glo 3/7 substrate in a 96-well plate in the dark for 1 h at room temperature. Luciferase activity was determined using a Lumicount Luminometer.

### Statistical analysis

For quantitative analyses, a two-tailed, unpaired Student *t* test was used to analyze differences in mean values between groups. All results were expressed as mean +/− standard deviations (SD) of the means. *P* values of <0.05 were considered significant.

## Supplementary information

Supplementary Table 1
