# Supplementary material for: A natural polymorphism in Zika virus NS2A protein responsible of virulence in mice
Source: Sci Rep. 2019 Dec 27;9:19968. doi: 10.1038/s41598-019-56291-4 (PMC6934710; doi:10.1038/s41598-019-56291-4)
Supplement: Supplementary file 1 — Supplementary Table 1 [file 41598_2019_56291_MOESM1_ESM.pdf]

A natural polymorphism in Zika virus NS2A protein responsible of virulence in mice

Gines Ávila-Pérez[1,*], Aitor Nogales[1,2,*], Jun-Gyu Park[1], Silvia Márquez-Jurado[3], Francisco J. Iborra[3], Fernando Almazan[3,&], and Luis Martínez-Sobrido[1,&]

[1]Department of Microbiology and Immunology, University of Rochester Medical Center, 601 Elmwood Avenue, Rochester, New York, 14642, USA.

[2]Center for Animal Health Research, INIA-CISA, 28130 Valdeolmos, Madrid, Spain.

[3]Department of Molecular and Cell Biology, Centro Nacional de Biotecnología (CNB-CSIC), Universidad Autonóma de Madrid, 3 Darwin Street, 28049 Madrid, Spain.

*These authors contributed equally

[&] Correspondence should be addressed:

luis_martinez@urmc.rochester.edu ; Tel.: (585) 276-4733

falmazan@cnb.csic.es ; Tel.: +34-91-585-5339

Supplementary Table 1. Differences between University of Rochester (UR) ZIKV-Paraiba and ZIKV-Paraiba reference sequence in Genbank (accession number KX280026). Nucleotides differences with more than 50% of frequency that result in amino acid changes are shown in italic. [a]Only quasispecies with more than 10% of frequency are shown. Rows highlighted in grey indicate amino acid changes. [b]Amino acid changes in ZIKV proteins.

Nucleotide sequence			Amino acid sequence				
Nucleotide position	Reference sequence	UR sequence[a]	Polyprotein position	Protein position[b]	Reference sequence	UR sequence	Protein
535	*A*	*A 33%*	*143*	*21*	*E*	*E 33%*	*prM*
		G 67%				*G 67%*	
693	*A*	*G 100%*	*196*	*74*	*T*	*A 100%*	*prM*
798	*T*	*C 100%*	*231*	*109*	*S*	*P 100%*	*prM*
1,646	A	A 81%	-	-	-	-	E
		G 19%					
2,178	C	C 65%	691	401	H	H 65%	E
		T 35%				Y 35%	
2,960	T	T 86%	-	-	-	-	NS1
		C 14%					
3,400	G	G 88%	1,098	304	S	S 88%	NS1
		A 12%				N 12%	
3,828	T	T 63%	1,241	95	F	F 63%	NS2A

		C 37%				L 37%	
3,895	*C*	*C 47%*<br>*T 53%*	*1,263*	*117*	*A*	*A 47%*<br>*V 53%*	*NS2A*
4,667	C	C 71%<br>T 29%	-	-	-	-	NS3
5,614	*T*	*T 27%*<br>*C 63%*	*1,836*	*334*	*M*	*M 27%*<br>*T 63%*	*NS3*
6,373	*A*	*G 100%*	*2,089*	*587*	*K*	*R 100%*	*NS3*
6,724	C	C 83%<br>T 17%	2,206	86	T	T 83%<br>I 17%	NS4A
7,136	T	T 68%	-	-	-	-	NS4B
